# Book Review: Fact and Fiction in Global Energy Policy. 15 Contentious Questions

**DOI:** 10.3389/fpsyg.2017.00292

**Published:** 2017-03-02

**Authors:** Goda Perlaviciute

**Affiliations:** Environmental Psychology, Faculty of Behavioural and Social Sciences, University of GroningenGroningen, Netherlands

**Keywords:** energy policy, energy contentions, values, common ground, ideologies

In the Paris Climate Agreement, countries worldwide committed to reduce greenhouse gas emissions by adopting more sustainable methods of energy use and supply. Yet, there are conflicting views on how to accomplish this goal and which energy sources, systems, and policies should be prioritized in a sustainable energy transition. In their book, Benjamin K. Sovacool, Marilyn A. Brown, and Scott V. Valentine present such diverse perspectives and explore possibilities to reach common ground.

## Diverse perspectives

The authors introduce globally important topics on climate change and sustainable energy policy as a series of *15 contentious questions*, and present two opposing points of view to each question. For example, both supporters and opponents give arguments to substantiate their position on the question whether shale gas is a bridge to a clean energy future. For each topic, the authors extensively review the arguments of each side, supported with scientific literature, policy analysis, and examples from the media. Intriguingly, the authors demonstrate that each topic could turn into a battlefield between different parties. The same outcomes of energy policy are seen positively by some people and negatively by others, and the conflicting parties are often not open to alternative arguments. For example, supporters claim that shale gas *is sustainable* because it is cleaner than other fossil fuels and can serve as a backup for renewables (e.g., when there is not enough sun or wind). Conversely, opponents argue that shale gas *is not sustainable* because it emits greenhouse gases and stalls investments in renewables. Indeed, research has shown that people evaluate energy developments in an overly positive or negative way: namely, as either having many benefits and few costs or many costs and few benefits (Finucane et al., 2000). The authors propose that such conflicting perspectives are driven by different ideologies. For example, some people think that technology will solve all environmental and energy problems (“technological optimist”) while other people think that technology is not the (only) solution and that people must also change their individual behaviors (“conscientious consumer”). However, it is not explicit in the book where such different ideologies stem from. Yet, research has shown that the way people evaluate different energy developments—and their associated costs and benefits—depends on how these energy developments impact their core values (Bidwell, 2013; De Groot et al., 2013; Perlaviciute and Steg, 2015). This could potentially explain the entrenched disagreements on environmental and energy problems, as well as sustainable energy policy.

Importantly, the authors argue that although disagreements about a sustainable energy transition are inevitable, they are not necessarily dysfunctional. While different parties perceive certain energy sources, systems, and policies as having either only positive or negative outcomes, in reality different energy developments have various positive *and* negative outcomes at the same time. The authors take different opinions equally into account, which enables them to propose potential solutions that better incorporate key concerns of different groups.

## Common ground

The discussion on each of the 15 questions ends with a synthesis of the arguments from both sides and their potential common ground to reconcile the divergent positions. For example, the implementation of shale gas can only be beneficial and legitimate if it is adequately governed, the risks are properly managed, and as long as the use of shale gas is planned as a bridge to renewable energy rather than a goal in itself. Crucially, the authors demonstrate that a sustainable energy transition requires compromises between the diverse perspectives.

The question remains, however, how to accomplish such common ground and compromises in practice. In the concluding chapter, the authors offer six maxims for policymakers, as well as to the general public, to better understand and manage controversial energy issues. These include: revealing competing interests and understanding how they are reflected in energy decisions (“Know the players”); staying informed about energy technologies and issues (“Inform yourself”); aiming for ethical and well-informed decisions to manage risks and uncertainty (“Be prudent about risk”); considering diverse interests in energy decisions (“Seek diversity and inclusivity”); becoming aware of values and ideologies that underlie different energy perspectives (“Practice self-reflection”); and seeing energy technology as a means to satisfy societal needs rather than a goal in itself (“Embrace technological agnosticism”). Yet, it is not clear how to successfully realize these steps and overcome potential barriers. For example, the authors acknowledge that staying informed may not lead to better decisions if people adhere mostly to information that supports their perspective and neglect or disqualify information that challenges their perspective. Furthermore, if people's values influence how they evaluate different energy developments (Bidwell, 2013; De Groot et al., 2013; Perlaviciute and Steg, 2015), the more salient question would be how to prioritize different values in decision making. The authors argue that the interests of different stakeholders—citizens included—should be represented and incorporated in decision making. Yet, in practice, it may be difficult to motivate the public to participate in the decision making process and to ensure that the different sides are equally represented (Irvin and Stansbury, 2004). Furthermore, when faced with competing perspectives, people may not seek compromises but rather take even more extreme sides, a phenomenon known as attitude polarization (Lord et al., 1979; Pomerantz et al., 1995; Kahan et al., 2012). The six maxims outlined in the book offer a starting point for systematically studying the conditions under which diverse perspectives can be represented and incorporated in decision making, facilitating a sustainable and societally acceptable energy transition.

This book is not a collection of facts about energy developments—its original character lies in bringing together diverse *subjective* perspectives on energy topics, which are influenced and even biased by people's ideologies and values. The authors demonstrate that it is important to carefully consider such diverse perspectives in order to find solutions that better integrate the interests of different groups. The book therefore urges policymakers and the general public to critically think on how to incorporate such diverse perspectives in a truly sustainable global energy policy.

## Author contributions

The author confirms being the sole contributor of this work and approved it for publication.

### Conflict of interest statement

The author declares that the research was conducted in the absence of any commercial or financial relationships that could be construed as a potential conflict of interest.
